# Exploring the Impacts of Meaning in Life, Character Strengths, and Social Connectedness on Affect and Achievement in Gifted Students

**DOI:** 10.3390/jintelligence14010007

**Published:** 2026-01-04

**Authors:** Paul Shing-fong Chan, Mantak Yuen, Jiahong Zhang

**Affiliations:** 1 Department of Psychology, The Education University of Hong Kong, Hong Kong 999077, China; 2 Department of Counselling and Psychology, Hong Kong Shue Yan University, Hong Kong 999077, China; 3 Centre for Advancement in Inclusive and Special Education, Faculty of Education, The University of Hong Kong, Hong Kong 999077, China; zjh2021@hku.hk

**Keywords:** gifted children, meaning in life, character strengths, social connectedness, positive affect, negative affect, academic achievement, Hong Kong

## Abstract

Understanding the factors that promote positive affect and achievement in gifted students is essential for supporting their holistic development and success. This study aimed to explore the relationship among meaning in life (presence and search), character strengths (creativity, perseverance, social intelligence), social connectedness, positive/negative affect, and perceived academic achievement among gifted students in Hong Kong, China. A total of 348 gifted students participated in this study, comprising 196 males and 152 females, aged 10 to 18 years. The students completed a cross-sectional online survey in August and September 2024. Mediation analyses were conducted using structural equation modeling with bootstrapping to estimate indirect effects. The results indicated significant indirect effects of meaning in life (presence and search) and character strengths (creativity, perseverance, social intelligence) on positive affect (β = 0.15 to 0.32, *p* < 0.05) and negative affect (β = −0.15 to −0.26, *p* < 0.05) via social connectedness, with small-to-moderate effect sizes. Additionally, significant indirect effects were observed for meaning in life and character strengths on perceived academic achievement via social connectedness (β = 0.13 to 0.20, *p* < 0.05), with small-to-moderate effect sizes. This research highlights the significant role of character strengths, meaning in life, and social connectedness in enhancing positive affect and perceived academic achievement, and reducing negative affect among gifted students.

## 1. Introduction

Gifted students demonstrate exceptional cognitive abilities and creative talents that distinguish them from their age-level peers in academic and intellectual domains. However, advanced cognitive capacities alone do not guarantee positive affect or high academic achievement. In fact, gifted students frequently encounter unique social, emotional, and psychological challenges, such as heightened sensitivity, perfectionism, and difficulties in social integration (Neihart et al., 2021; J. S. Peterson, 2009). These challenges highlight the need for research that extends beyond intellectual aptitude to identify factors contributing to the holistic well-being and success of gifted students.

Recent research in positive psychology underscores the significance of meaning in life, character strengths, and social connectedness as critical contributors to emotional well-being and achievement among youth (Park, 2004; Seligman et al., 2009). The PERMA model provides a robust theoretical foundation for integrating meaning in life, character strengths, social connectedness, affect, and academic achievement (Seligman, 2011). PERMA encompasses Positive Emotions (aligning with positive/negative affect), Engagement (supported by character strengths like creativity, perseverance, and social intelligence), Relationships (reflected in social connectedness), Meaning (directly tied to presence and search for meaning in life), and Accomplishment (encompassing perceived academic achievement). These elements are interconnected and have been supported by research studies. For instance, meaning in life has been linked to enhanced positive affect and improved adaptive functioning among adolescents (Kiang & Fuligni, 2010; Tavernier & Willoughby, 2012). Similarly, character strengths such as creativity, perseverance, and social intelligence are linked to academic success and psychological resilience (Azañedo et al., 2020; Duckworth et al., 2007; Gajda et al., 2017). Social connectedness, defined as a sense of belonging and having positive relationships with others, is particularly salient for gifted students, who may struggle to form meaningful peer connections due to their distinctive interests and abilities (Machů & Červinková, 2014).

Nevertheless, the mechanisms by which meaning in life and character strengths influence affect and achievement in gifted students remain under-explored, particularly within Asian educational contexts. In Hong Kong, China, where academic competition is intense and expectations are high, understanding how social factors interact is crucial for informing effective educational and counseling interventions for gifted youth (Dai & Kuo, 2015).

The construct of meaning in life, encompassing both the presence of meaning and the search for meaning, has been increasingly recognized as an important aspect of adolescent development (Tavernier & Willoughby, 2012). Among gifted students, who often contend with existential questions and heightened sensitivity, the presence of meaning may play a particularly vital role in their emotional adjustment and resilience (Neihart et al., 2021). However, the search for meaning can be a double-edged sword, sometimes associated with distress or uncertainty, but also potentially motivating personal growth (Steger et al., 2008). There is a dearth of studies directly examining how meaning in life relates to affect or academic outcomes among gifted populations, especially within the context of East Asian educational cultures.

Character strengths, as outlined in the Values in Action (VIA) framework, represent positive traits reflected in thoughts, feelings, and behaviors (C. Peterson & Seligman, 2004). Some research studies highlighted the importance of creativity, perseverance, and social intelligence among gifted youth (Machů & Červinková, 2014; Runco, 1993; Shi & Ahn, 2015). Creativity is often a hallmark of giftedness and is linked with academic and extracurricular achievement (Runco, 1993). Perseverance predicts long-term academic success and is associated with resilience when facing academic and personal challenges (Duckworth et al., 2007). Social intelligence, the capacity to navigate social environments and relationships effectively, is essential for peer acceptance but may be an area of difficulty for some gifted students, who can feel isolated due to their unique interests and abilities (Machů & Červinková, 2014). Research suggests that character strengths are positively associated with well-being and achievement in adolescents (Weber & Harzer, 2022), but their specific roles and mechanisms among gifted students require further clarification.

Social connectedness, defined as the sense of belonging and the quality of interpersonal relationships, is a foundational aspect of adolescent well-being (Baumeister & Leary, 1995). For gifted students, positive peer relationships can serve as a buffer against the social-emotional challenges they often face (Mendaglio & Peterson, 2007). Conversely, lack of social connectedness can contribute to feelings of loneliness, anxiety, and depression (J. S. Peterson, 2009). Social connectedness has also been linked with academic engagement and motivation (Wentzel, 2005). In collectivist cultures such as Hong Kong, where group harmony and social integration are highly valued, the importance of social connectedness may be even more pronounced. However, gifted students may experience difficulties finding peers with similar interests or abilities, which can impact their sense of belonging and emotional well-being (J. S. Peterson, 2009).

Emerging research suggests that meaning in life and character strengths may enhance emotional well-being and achievement directly, and also indirectly through fostering social connectedness (Donohoe & Greene, 2009; Zuo et al., 2024). For instance, students with a strong sense of meaning and well-developed character strengths may be more likely to form and sustain positive relationships, thereby increasing their sense of belonging and social support (Machell et al., 2015). In turn, social connectedness has been shown to mediate the relationship between individual psychological assets and outcomes such as affect and academic performance (Donohoe & Greene, 2009; Zuo et al., 2024). Despite these promising associations, there is a paucity of research examining these mediation pathways specifically in gifted student populations or in East Asian contexts.

Hong Kong’s highly competitive academic environment places considerable pressure on students, including those identified as gifted. The interplay between meaning in life, character strengths, and social factors may be especially complex in this setting, where high achievement is culturally emphasized but social and emotional needs may be overlooked (Machů & Červinková, 2014). Understanding how meaning in life, character strengths, and social connectedness jointly influence affect and academic outcomes is critical for designing effective support programs for gifted youth in Hong Kong and similar contexts.

While previous studies have established the importance of meaning in life, character strengths, and social connectedness for general adolescent populations, there is a dearth of studies exploring their interrelations and mediating mechanisms among gifted students, particularly in non-Western societies. This study addressed these research gaps by examining the direct and indirect effects of meaning in life and character strengths on affect and perceived academic achievement, with social connectedness as a mediator, in a sample of gifted students in Hong Kong. In this study, there were five hypotheses:

1. Meaning in life (presence and search), character strengths (creativity, perseverance, social intelligence), and social connectedness would be positively related to positive affect and perceived academic achievement, and negatively related to negative affect among gifted students in Hong Kong.
2. Social connectedness would mediate the relationship between meaning in life (presence and search) and affect, such that higher meaning in life would indirectly predict higher positive affect and lower negative affect via greater social connectedness.
3. Social connectedness would mediate the relationship between meaning in life (presence and search) and perceived academic achievement, such that higher meaning in life would indirectly predict higher perceived academic achievement via greater social connectedness.
4. Social connectedness would mediate the relationship between character strengths (creativity, perseverance, social intelligence) and affect, such that stronger character strengths would indirectly predict higher positive affect and lower negative affect via greater social connectedness.
5. Social connectedness would mediate the relationship between character strengths (creativity, perseverance, social intelligence) and perceived academic achievement, such that stronger character strengths would indirectly predict higher perceived academic achievement via greater social connectedness.

## 2. Materials and Methods

### 2.1. Participants and Data Collection

Participants were recruited from Hong Kong primary and secondary schools via The Hong Kong Academy for Gifted Education. The Academy identified gifted students for admission by being nominated by school principals, teachers, parents or students themselves, by testing or from evidence of the students’ ability from their academic record at school or competitions. Additional evidence of creativity, talent performance in school and outside school, and task commitment was also taken into account. The participants were gifted in at least one of the following areas: leadership, mathematics, language, science, music, visual arts, sports, culinary arts, artificial intelligence, or engineering.

A total of 348 students completed the survey, of which 56.3% (*n* = 196) were males. They were aged from 10 to 18 years. Approximately half were Secondary 1–3 students (49.5%, *n* = 172), and a quarter of students were Primary 4–6 students (26.4%, *n* = 92) and Secondary 4–6 students (24.1%, *n* = 104). Nearly half (55.5%, *n* = 193) reported that their academic performance was the top 10% among their classmates. The majority of their fathers (67.6%, *n* = 235) and mothers (64.7%, *n* = 225) received tertiary education, had siblings (61.2%, *n* = 213), and had English as a medium of instruction in school (62.1%, *n* = 216) (Table 1).

Approximately 80.2% (*n* = 279) reported that they had at least one type of talent, including leadership (56.9%, *n* = 198), mathematics (49.4%, *n* = 172), language (51.7%, *n* = 180), science (56.6%, *n* = 197), music (43.7%, *n* = 152), visual arts (35.9%, *n* = 125), sports (29.0%, *n* = 101), culinary arts (23.6%, *n* = 82), artificial intelligence (36.8%, *n* = 128), and engineering (33.9%, *n* = 118) (Table 1).

Data were collected via an anonymous online survey in August and September 2024. Informed consent was obtained from participants and their parents/guardians, and the study was approved by The Human Research Ethics Committee, The University of Hong Kong (Ref. no.: EA1904021).

### 2.2. Measures

#### 2.2.1. Meaning in Life

The Meaning in Life Questionnaire was used, which consists of two subscales, namely presence and search for meaning (Steger et al., 2006). Each subscale consists of five items and participants rated on a 7-point Likert scale (1 = completely incorrect to 7 = completely correct). Sample items include “I understand the meaning of my life” (presence) and “I am searching for something that makes my life feel meaningful” (search). The internal consistency of presence and search for meaning, measured by Cronbach’s alpha, was 0.86 and 0.90, respectively, in this study. Descriptive statistics for the presence and search subscales were computed by summing the individual items and the possible range of scores for presence and search was from 5 to 35. 

#### 2.2.2. Character Strengths

Three character strengths, including creativity, perseverance, and social intelligence, were measured using subscales from the VIA of Strengths for Youth (Park & Peterson, 2006). Each subscale consists of four items and participants were instructed to rate on a 5-point Likert scale (1 = not at all like me to 5 = extremely like me). Sample items include “I enjoy creating novel and different things” (creativity), “Even when faced with many challenges, I still complete all my assignments” (perseverance), and “I am able to avoid conflicts or problems with others” (social intelligence). The internal consistency of creativity, perseverance, and social intelligence, measured by Cronbach’s alpha, was 0.88, 0.84 and 0.71, respectively, in this study. The scores of creativity, perseverance, and social intelligence were computed by summing individual items. The possible range of scores for creativity, perseverance, and social intelligence was from 4 to 20.

#### 2.2.3. Social Connectedness

Participants’ degree of social connectedness was measured with the Hemingway—Measure of Adolescent Connectedness (Karcher & Sass, 2010). It comprises four subscales gauging participants’ connectedness to various areas, including parents, school, peers, and teachers. Each subscale contains four or five items and there are 17 items in total. Participants were asked to rate on a 5-point Likert scale (1 = completely untrue to 5 = completely true). Sample items include “I enjoy the time I spend with my parents” (parents), “I study hard at school” (school), “I enjoy working with my classmates.” (peers), and “I try to get along well with my teachers” (teachers). The internal consistency of all subscales, measured by Cronbach’s alpha ranged from 0.81 to 0.90 in this study. The scores of the total social connectedness scale and its parents, school, peers, and teachers subscales were computed by summing the individual items. The possible range of scores for total social connectedness was from 17 to 85, while it was from 4 to 20 for parents, peers, and teachers, and from 5 to 25 for school.

#### 2.2.4. Affect

Positive and negative affect were assessed with the Positive and Negative Affect Schedule for the participants (Thompson, 2007). Both affects consist of five items and participants rated each item on a 5-point scale (1 = never to 5 = always). Sample items include “determined” and “attentive” for positive affect, and “upset” and “nervous” for negative affect. The internal consistency of positive and negative affect, measured by Cronbach’s alpha, was 0.60 and 0.80, respectively, in this study. For positive affect, after removing one item (“alert”), the Cronbach’s alpha increased to 0.68. Therefore, this item was excluded in the mediation analysis. The scores of the positive affect and negative affect subscales were computed by summing the individual items. The possible range of scores for positive and negative affect was from 4 to 20 and from 5 to 25, respectively.

#### 2.2.5. Perceived Academic Achievement

Perceived academic achievement was assessed via a self-rated single item comparing performance to classmates—“Compared to other classmates in your class, your score on the most recent exam was…” The participants rated this item on a five-point scale (1 = top 10%, 2 = between 10–40%, 3 = between 40–60%, 4 = between 60–90%, 5 = bottom 10%).

### 2.3. Data Analysis

Mediation analyses were conducted using structural equation modelling (SEM) with maximum likelihood estimation and bootstrapping (2000 resamples) to estimate bias-corrected 95% confidence intervals (CIs) for indirect effects (Ledermann et al., 2025). In these analyses, meaning in life (presence, search) and character strengths (creativity, perseverance, social intelligence) served as independent variables, affect (positive, negative) and perceived academic achievement as the dependent variables, and social connectedness (parents, school, peers, teachers) as the mediator. Effect sizes were reported as standardized coefficients (β). The models were evaluated by examining the following model fit indexes: (1) comparative fit index (CFI), (2) incremental fit index (IFI), (3) nonnormed fit index (NNFI), (4) root mean square error of approximation (RMSEA). A model fit was considered good when the values of CFI, IFI, and NNFI were equal to or above 0.90 and RMSEA was lower than 0.08.

## 3. Results

### 3.1. Descriptive Statistics of the Study Variables

Descriptive statistics of the study variables were presented in Table 2. For meaning in life, scores for presence ranged from 5 to 35 (mean (M) = 24.52, standard deviation (SD) = 6.37), while search ranged from 5 to 35 (M = 26.61, SD = 6.05). Regarding character strengths, creativity scores ranged from 4 to 20 (M = 14.26, SD = 3.39), perseverance from 4 to 20 (M = 14.65, SD = 3.43), and social intelligence from 6 to 20 (M = 14.04, SD = 3.08). For total social connectedness, scores ranged from 27 to 85 (M = 67.91, SD = 9.99). For parents, scores ranged from 4 to 20 (M = 16.00, SD = 3.22). For school, scores ranged from 8 to 25 (M = 20.35, SD = 3.19). For peers, scores ranged from 6 to 20 (M = 15.11, SD = 3.01). For teachers, scores ranged from 4 to 20 (M = 16.45, SD = 2.66). For positive affect, scores ranged from 4 to 20 (M = 13.99, SD = 2.60), whilst scores for negative affect ranged from 5 to 25 (M = 13.45, SD = 3.43).

### 3.2. Impacts of Meaning in Life, Character Strengths, and Social Connectedness on Affect and Perceived Academic Achievement

The impacts of meaning in life, character strengths, and social connectedness on affect and perceived academic achievement were presented below. All mediation models had good model fit, i.e., CFI > 0.9, IFI > 0.9, NNFI > 0.9, RMSEA < 0.08 (Table 3), except two models showed slightly lower NNFI values: social intelligence -> social connectedness -> positive affect (NNFI = 0.896), and social intelligence -> social connectedness -> negative affect (NNFI = 0.894). These marginal deviations were considered acceptable in complex SEM mediation analyses, as they remain close to the cutoff and were supported by strong CFI, IFI, and RMSEA values, indicating robust overall model fit for exploratory research in psychological constructs (Hu & Bentler, 1999). No substantial misfit was observed that would compromise the interpretation of indirect effects.

#### 3.2.1. Impacts of Meaning in Life and Social Connectedness on Affect

The indirect effect of presence on positive affect via social connectedness was significant and positive (β = 0.32, *p* < 0.001, 95% CI: 0.22, 0.46), indicating a moderate effect size. The direct effect was also positive and significant (β = 0.27, *p* = 0.002, 95% CI: 0.09, 0.43). For search, the indirect effect was significant and positive (β = 0.27, *p* < 0.001, 95% CI: 0.19, 0.38), with a small-to-moderate effect size, while the direct effect was not significant (β = 0.07, *p* = 0.29, 95% CI: −0.07, 0.21) (Table 4 and Table 5; Supplementary Figures S1 and S2).

The indirect effect of presence on negative affect via social connectedness was significant and negative (β = −0.19, *p* = 0.002, 95% CI: −0.34, −0.06), indicating a small-to-moderate effect size. The direct effect was not significant (β = −0.09, *p* = 0.35, 95% CI: −0.31, 0.11). For search, the indirect effect was significant and negative (β = −0.15, *p* < 0.001, 95% CI: −0.24, −0.08), with a small effect size, while the direct effect was not significant (β = −0.01, *p* = 0.94, 95% CI: −0.17, 0.14) (Table 6 and Table 7; Supplementary Figures S3 and S4).

#### 3.2.2. Impacts of Meaning in Life and Social Connectedness on Perceived Academic Achievement

The indirect effect of presence on perceived academic achievement via social connectedness was significant and positive (β = 0.20, *p* < 0.001, 95% CI: 0.13, 0.30), indicating a small-to-moderate effect size. The direct effect was not significant (β = −0.10, *p* = 0.16, 95% CI: −0.23, 0.03). For search, the indirect effect was significant and positive (β = 0.14, *p* < 0.001, 95% CI: 0.08, 0.20), with a small effect size, while the direct effect was significant and negative (β = −0.14, *p* = 0.004, 95% CI: −0.23, −0.04) (Table 8 and Table 9; Supplementary Figures S5 and S6).

#### 3.2.3. Impacts of Character Strengths and Social Connectedness on Affect

The indirect effect of creativity on positive affect via social connectedness was significant and positive (β = 0.20, *p* < 0.001, 95% CI: 0.14, 0.30), indicating a small-to-moderate effect size. The direct effect was also positive and significant (β = 0.54, *p* < 0.001, 95% CI: 0.31, 0.73). For perseverance, the indirect effect was significant and positive (β = 0.15, *p* = 0.008, 95% CI: 0.05, 0.27), with a small effect size, and the direct effect was positive and significant (β = 0.72, *p* < 0.001, 95% CI: 0.57, 0.87). For social intelligence, the indirect effect was significant and positive (β = 0.27, *p* < 0.001, 95% CI: 0.18, 0.40), with a small-to-moderate effect size, and the direct effect was positive and significant (β = 0.40, *p* = 0.002, 95% CI: 0.21, 0.56) (Table 10, Table 11 and Table 12; Supplementary Figures S7–S9).

The indirect effect of creativity on negative affect via social connectedness was significant and negative (β = −0.17, *p* < 0.001, 95% CI: −0.28, −0.09), indicating a small effect size. The direct effect was not significant (β = −0.02, *p* = 0.84, 95% CI: −0.17, 0.14). For perseverance, the indirect effect was significant and negative (β = −0.26, *p* < 0.001, 95% CI: −0.43, −0.14), with a small-to-moderate effect size, and the direct effect was not significant (β = 0.10, *p* = 0.43, 95% CI: −0.13, 0.30). For social intelligence, the indirect effect was significant and negative (β = −0.21, *p* < 0.001, 95% CI: −0.38, −0.11), with a small-to-moderate effect size, and the direct effect was not significant (β = −0.03, *p* = 0.72, 95% CI: −0.22, 0.17) (Table 13, Table 14 and Table 15; Supplementary Figures S10–S12).

#### 3.2.4. Impacts of Character Strengths and Social Connectedness on Perceived Academic Achievement

The indirect effect of creativity on perceived academic achievement via social connectedness was significant and positive (β = 0.13, *p* < 0.001, 95% CI: 0.07, 0.21), indicating a small effect size. The direct effect was not significant (β = −0.02, *p* = 0.84, 95% CI: −0.15, 0.13). For perseverance, the indirect effect was significant and positive (β = 0.13, *p* = 0.02, 95% CI: 0.02, 0.22), with a small effect size, while the direct effect was not significant (β = 0.13, *p* = 0.11, 95% CI: −0.03, 0.33). For social intelligence, the indirect effect was significant and positive (β = 0.19, *p* < 0.001, 95% CI: 0.10, 0.30), with a small-to-moderate effect size, while the direct effect was not significant (β = −0.08, *p* = 0.40, 95% CI: −0.25, 0.11) (Table 16, Table 17 and Table 18; Supplementary Figures S13–S15).

## 4. Discussion

There are two main strengths of this study. First, to the best of our knowledge, this is one of the first studies investigating the interrelationships among meaning in life, character strengths, social connectedness, positive and negative affect, and perceived academic achievement, as well as the mediating role of social connectedness in the pathways through which psychological resources contribute to emotional well-being and academic achievement among gifted students in Hong Kong, China. Second, a diverse sample of gifted students participated in this study. The gifted students were from a range of grade levels, spanning Primary Four to Secondary Six, and were talented in diverse areas (e.g., leadership, mathematics, science, arts, AI, engineering).

Our findings confirm that both the presence of and search for meaning in life were associated with higher positive affect and perceived academic achievement, as well as lower negative affect among gifted students via social connectedness, with small-to-moderate indirect effects via social connectedness (β ranging from 0.14 to 0.32 for positive affect and perceived academic achievement; β from −0.15 to −0.19 for negative affect). Character strengths, i.e., creativity, perseverance, and social intelligence, also predicted greater positive affect and higher perceived academic achievement, while correlating with reduced negative affect, again with small-to-moderate indirect effects through social connectedness (β ranging from 0.13 to 0.27 for positive affect and perceived academic achievement; β from −0.17 to −0.26 for negative affect). These bootstrapped indirect effects were robust, as evidenced by non-zero 95% CIs, highlighting social connectedness as a key mechanism (Ledermann et al., 2025). Gifted students who felt more socially connected were more likely to experience the benefits of meaning in life and character strengths, suggesting that social belonging amplifies the positive effects of these internal assets.

Theoretically, this study further enriches positive psychology frameworks, particularly the PERMA model proposed by Seligman (2011), which emphasizes positive emotions, engagement, relationships, meaning, and accomplishment as pillars of well-being. By demonstrating that meaning in life and character strengths influence affect and achievement through social connectedness, our results extended this model to gifted adolescents, highlighting how relationships (a key PERMA element) served as a mediator for meaning and strengths to foster positive emotions and accomplishment.

Practically, the study findings underscore the importance of adopting a holistic approach to gifted education, one that recognizes the interplay between internal strengths and external social factors. The consistent indirect effects through social connectedness (with effect sizes up to β = 0.32) were consistent with theories that emphasize belonging as a fundamental human motivation (Baumeister & Leary, 1995) and as particularly salient for gifted students, who often report feelings of social isolation or difference (Neihart et al., 2021; J. S. Peterson, 2009). Educational programs for gifted students should therefore not only foster meaning-making and character development, but also actively promote social integration and peer relationships. Initiatives such as collaborative projects, mentoring, and group problem-solving activities can enhance social connectedness and, as our results suggest, maximize the emotional and academic benefits associated with meaning in life and character strengths. This is especially relevant in the context of Hong Kong’s competitive academic culture, where the pressure to achieve can sometimes lead to social withdrawal or stress (Wong & Ying, 2006). Furthermore, beyond educational settings, the indirect effects underscore practical implications for parental involvement in supporting gifted children’s development. In the context of Hong Kong’s high-pressure academic environment, where gifted youth may face isolation, parental involvement, such as through supportive communication, encouragement of talent exploration, and facilitation of social opportunities, can serve as a protective factor, buffering emotional challenges (Morawska & Sanders, 2008). Interventions should empower parents with strategies to enhance connectedness, such as family workshops on meaning-making discussions, to optimize the holistic development of gifted adolescents.

On a broader scale, the findings advocate for policy-level changes in gifted education systems, particularly in regions like Hong Kong where academic excellence is usually prioritized over socio-emotional development. Policymakers could integrate social connectedness metrics into gifted program evaluations, ensuring that curricula include components for building social connectedness, meaning in life and character strengths (Dai & Kuo, 2015). Additionally, government may consider allocating resources for teacher training in positive psychology (Pfeiffer, 2012). Such policies would promote equity, ensuring that gifted education addresses not just intellectual but also emotional and social needs for long-term societal contributions.

For cultural considerations, our research was conducted within the sociocultural environment of Hong Kong, where collectivist values and academic achievement are highly emphasized. The pronounced indirect effects of social connectedness in mediating emotional well-being and academic achievement in our sample may reflect these cultural priorities, supporting earlier findings that social harmony and connectedness are particularly important in East Asian educational contexts (Markus & Kitayama, 1991).

Nonetheless, there are several limitations in this study. First, the cross-sectional design prevents cause-and-effect relationships. Longitudinal studies are needed to clarify the directionality of independent and dependent variables. Second, the data were self-reported, which may introduce recall, response, or social desirability biases. Third, our sample was limited to gifted students in Hong Kong, and hence findings may not generalize to other populations or cultural contexts.

Future research could examine the relationships suggested in this study longitudinally, explore interventions that foster social connectedness, or compare gifted and non-gifted student groups, and/or across diverse cultures. Qualitative studies could also offer deeper insights into the lived experiences of gifted youth regarding meaning in life, character strengths, and social connectedness.

## 5. Conclusions

In summary, our study highlights the critical role of social connectedness in linking meaning in life and character strengths to emotional well-being and academic self-perceptions among gifted students, as evidenced by significant indirect effects with small-to-moderate effect sizes based on bootstrapped analyses. There are theoretical and practical implications in this study. Theoretically, this study extends Seligman’s PERMA model to gifted students, demonstrating how social connectedness channeled the indirect influences of meaning in life and character strengths’ effects on affect and perceived academic achievement. Practically, it calls for holistic gifted education with social integration programs, parental involvement strategies, and policy reforms prioritizing socio-emotional needs.

## Figures and Tables

**Table 1:** Participants’ background characteristics (*n* = 348).

	*n*	%
Gender		
Male	196	56.3
Female	152	43.7
Grade		
Primary 4–6	92	26.4
Secondary 1–3	172	49.4
Secondary 4–6	84	24.1
Perceived academic achievement in the class		
Top 10%	193	55.5
Between 10–40%	116	33.3
Between 40–60%	28	8.0
Between 60–90%	7	2.0
Bottom 10%	4	1.1
Father’s education level		
Primary or below	5	1.4
Secondary	107	30.7
College or above	236	67.8
Mother’s education level		
Primary or below	5	1.4
Secondary	118	33.9
College or above	225	64.7
Having siblings		
Yes	213	61.2
No	135	38.8
Medium of instruction		
Chinese	131	37.6
English	217	62.4
Having at least one kind of talent		
Yes	279	80.2
No	69	19.8
Talents		
Leadership	198	56.9
Mathematics	172	49.4
Language	180	51.7
Science	197	56.6
Music	152	43.7
Visual arts	125	35.9
Sports	101	29.0
Culinary arts	82	23.6
Artificial intelligence	128	36.8
Engineering	118	33.9

**Table 2:** Descriptive statistics of the study variables (*n* = 348).

Study Variables	Possible Range	Minimum	Maximum	M	SD
Meaning in Life					
Presence	5–35	5	35	24.52	6.37
Search	5–35	5	35	26.61	6.05
Character strengths					
Creativity	4–20	4	20	14.26	3.39
Perseverance	4–20	4	20	14.65	3.43
Social intelligence	4–20	6	20	14.04	3.08
Social connectedness	17–85	27	85	67.91	9.99
Parents	4–20	4	20	16.00	3.22
School	5–25	8	25	20.35	3.19
Peers	4–20	6	20	15.11	3.01
Teachers	4–20	4	20	16.45	2.66
Affect					
Positive	4–20	4	20	13.99	2.60
Negative	5–25	5	25	13.45	3.43

M: mean; SD: standard deviation.

**Table 3:** Indices of model fit.

Mediation Model	CFI	IFI	NNFI	RMSEA
presence → social connectedness → positive affect	0.913	0.913	0.903	0.065
search → social connectedness → positive affect	0.917	0.917	0.908	0.063
presence → social connectedness → negative affect	0.911	0.909	0.905	0.067
search → social connectedness → negative affect	0.910	0.915	0.909	0.070
presence → social connectedness → perceived academic achievement	0.918	0.916	0.910	0.066
search → social connectedness → perceived academic achievement	0.923	0.928	0.913	0.067
creativity → social connectedness → positive affect	0.907	0.907	0.908	0.063
perseverance → social connectedness → positive affect	0.906	0.907	0.903	0.067
social intelligence → social connectedness → positive affect	0.908	0.912	0.896	0.068
creativity → social connectedness → negative affect	0.900	0.901	0.903	0.069
perseverance → social connectedness → negative affect	0.903	0.907	0.906	0.071
social intelligence → social connectedness → negative affect	0.907	0.913	0.894	0.075
creativity → social connectedness → perceived academic achievement	0.923	0.926	0.913	0.068
perseverance → social connectedness → perceived academic achievement	0.910	0.909	0.904	0.071
social intelligence → social connectedness → perceived academic achievement	0.908	0.909	0.905	0.077

CFI: comparative fit index; IFI: incremental fit index; NNFI: nonnormed fit index; RMSEA: root mean square error of approximation.

**Table 4:** Impacts of meaning in life—presence and social connectedness on positive affect.

	β	95% CI	*p* Value
**Path coefficients**			
presence → social connectedness (a)	0.61	---	<0.001
social connectedness → positive affect (b)	0.53	---	<0.001
presence → positive affect (c)	0.27	---	0.002
**Effects sizes**			
Indirect effects (=a × b)	0.32	0.22, 0.46	<0.001
Direct effects (c)	0.27	0.09, 0.43	0.002

CI: confidence interval.

**Table 5:** Impacts of meaning in life—search and social connectedness on positive affect.

	β	95% CI	*p* Value
**Path coefficients**			
search → social connectedness (a)	0.41	---	<0.001
social connectedness → positive affect (b)	0.67	---	0.002
search → positive affect (c)	0.07	---	0.29
**Effects sizes**			
Indirect effects (=a × b)	0.27	0.19, 0.38	<0.001
Direct effects (c)	0.07	−0.07, 0.21	0.29

CI: confidence interval.

**Table 6:** Impacts of meaning in life—presence and social connectedness on negative affect.

	β	95% CI	*p* Value
**Path coefficients**			
presence → social connectedness (a)	0.61	---	<0.001
social connectedness → negative affect (b)	−0.31	---	0.002
presence → negative affect (c)	−0.09	---	0.35
**Effects sizes**			
Indirect effects (=a × b)	−0.19	−0.34, −0.06	0.002
Direct effects (c)	−0.09	−0.31, 0.11	0.35

CI: confidence interval.

**Table 7:** Impacts of meaning in life—search and social connectedness on negative affect.

	β	95% CI	*p* Value
**Path coefficients**			
search → social connectedness (a)	0.41	---	<0.001
social connectedness → negative affect (b)	−0.36	---	<0.001
search → negative affect (c)	−0.01	---	0.94
**Effects sizes**			
Indirect effects (=a × b)	−0.15	−0.24, −0.08	<0.001
Direct effects (c)	−0.01	−0.17, 0.14	0.94

CI: confidence interval.

**Table 8:** Impacts of meaning in life—presence and social connectedness on perceived academic achievement.

	β	95% CI	*p* Value
**Path coefficients**			
presence → social connectedness (a)	0.60	---	<0.001
social connectedness → perceived academic achievement (b)	0.33	---	<0.001
presence → perceived academic achievement (c)	−0.10	---	0.16
**Effects sizes**			
Indirect effects (=a × b)	0.20	0.13, 0.30	<0.001
Direct effects (c)	−0.10	−0.23, 0.03	0.16

CI: confidence interval.

**Table 9:** Impacts of meaning in life—search and social connectedness on perceived academic achievement.

	β	95% CI	*p* Value
**Path coefficients**			
search → social connectedness (a)	0.41	---	<0.001
social connectedness → perceived academic achievement (b)	0.33	---	<0.001
search → perceived academic achievement (c)	−0.14	---	0.004
**Effects sizes**			
Indirect effects (=a × b)	0.14	0.08, 0.20	<0.001
Direct effects (c)	−0.14	−0.23, −0.04	0.004

CI: confidence interval.

**Table 10:** Impacts of creativity and social connectedness on positive affect.

	β	95% CI	*p* Value
**Path coefficients**			
creativity → social connectedness (a)	0.47	---	<0.001
social connectedness → positive affect (b)	0.43	---	<0.001
creativity → positive affect (c)	0.54	---	<0.001
**Effects sizes**			
Indirect effects (=a × b)	0.20	0.14, 0.30	<0.001
Direct effects (c)	0.54	0.31, 0.73	<0.001

CI: confidence interval.

**Table 11:** Impacts of perseverance and social connectedness on positive affect.

	β	95% CI	*p* Value
**Path coefficients**			
perseverance → social connectedness (a)	0.64	---	<0.001
social connectedness → positive affect (b)	0.23	---	0.009
perseverance → positive affect (c)	0.72	---	<0.001
**Effects sizes**			
Indirect effects (=a × b)	0.15	0.05, 0.27	0.008
Direct effects (c)	0.72	0.57, 0.87	<0.001

CI: confidence interval.

**Table 12:** Impacts of social intelligence and social connectedness on positive affect.

	β	95% CI	*p* Value
**Path coefficients**			
social intelligence → social connectedness (a)	0.57	---	<0.001
social connectedness → positive affect (b)	0.48	---	<0.001
social intelligence → positive affect (c)	0.40	---	0.002
**Effects sizes**			
Indirect effects (=a × b)	0.27	0.18, 0.40	<0.001
Direct effects (c)	0.40	0.21, 0.56	0.002

CI: confidence interval.

**Table 13:** Impacts of creativity and social connectedness on negative affect.

	β	95% CI	*p* Value
**Path coefficients**			
creativity → social connectedness (a)	0.48	---	0.002
social connectedness → negative affect (b)	−0.36	---	<0.001
creativity → negative affect (c)	−0.02	---	0.84
**Effects sizes**			
Indirect effects (=a × b)	−0.17	−0.28, −0.09	<0.001
Direct effects (c)	−0.02	−0.17, 0.14	0.84

CI: confidence interval.

**Table 14:** Impacts of perseverance and social connectedness on negative affect.

	β	95% CI	*p* Value
**Path coefficients**			
perseverance → social connectedness (a)	0.63	---	0.002
social connectedness → negative affect (b)	−0.42	---	<0.001
perseverance → negative affect (c)	0.10	---	0.43
**Effects sizes**			
Indirect effects (=a × b)	−0.26	−0.43, −0.14	<0.001
Direct effects (c)	0.10	−0.13, 0.30	0.43

CI: confidence interval.

**Table 15:** Impacts of social intelligence and social connectedness on negative affect.

	β	95% CI	*p* Value
**Path coefficients**			
social intelligence → social connectedness (a)	0.59	---	<0.001
social connectedness → negative affect (b)	−0.35	---	<0.001
social intelligence → negative affect (c)	−0.03	---	0.72
**Effects sizes**			
Indirect effects (=a × b)	−0.21	−0.38, −0.11	<0.001
Direct effects (c)	−0.03	−0.22, 0.17	0.72

**Table 16:** Impacts of creativity and social connectedness on perceived academic achievement.

	β	95% CI	*p* Value
**Path coefficients**			
creativity → social connectedness (a)	0.47	---	0.002
social connectedness → perceived academic achievement (b)	0.28	---	<0.001
creativity → perceived academic achievement (c)	−0.02	---	0.84
**Effects sizes**			
Indirect effects (=a × b)	0.13	0.07, 0.21	<0.001
Direct effects (c)	−0.02	−0.15, 0.13	0.84

CI: confidence interval.

**Table 17:** Impacts of perseverance and social connectedness on perceived academic achievement.

	β	95% CI	*p* Value
**Path coefficients**			
perseverance → social connectedness (a)	0.63	---	<0.001
social connectedness → perceived academic achievement (b)	0.20	---	0.02
perseverance → perceived academic achievement (c)	0.13	---	0.11
**Effects sizes**			
Indirect effects (=a × b)	0.13	0.02, 0.22	0.02
Direct effects (c)	0.13	−0.03, 0.33	0.11

CI: confidence interval.

**Table 18:** Impacts of social intelligence and social connectedness on perceived academic achievement.

	β	95% CI	*p* Value
**Path coefficients**			
social intelligence → social connectedness (a)	0.58	---	<0.001
social connectedness → perceived academic achievement (b)	0.32	---	<0.001
social intelligence → perceived academic achievement (c)	−0.08	---	0.40
**Effects sizes**			
Indirect effects (=a × b)	0.19	0.10, 0.30	<0.001
Direct effects (c)	−0.08	−0.25, 0.11	0.40

CI: confidence interval.

## Data Availability

The data presented in this study are available from the corresponding author upon request. The data are not publicly available as they contain personal behaviors.

## Supplementary Materials

The following supporting information can be downloaded at https://www.mdpi.com/article/10.3390/jintelligence14010007/s1: Supplementary Figures: Impacts of Meaning in Life, Character Strengths, and Social Connectedness on Affect and Achievement in Gifted Students.
