# Supplementary material for: Exploring the Impacts of Meaning in Life, Character Strengths, and Social Connectedness on Affect and Achievement in Gifted Students
Source: J Intell. 2026 Jan 4;14(1):7. doi: 10.3390/jintelligence14010007 (PMC12843387; doi:10.3390/jintelligence14010007)
Supplement: Supplementary file 1 [file jintelligence-14-00007-s001.zip › jintelligence-4001609-supplementary.pdf]

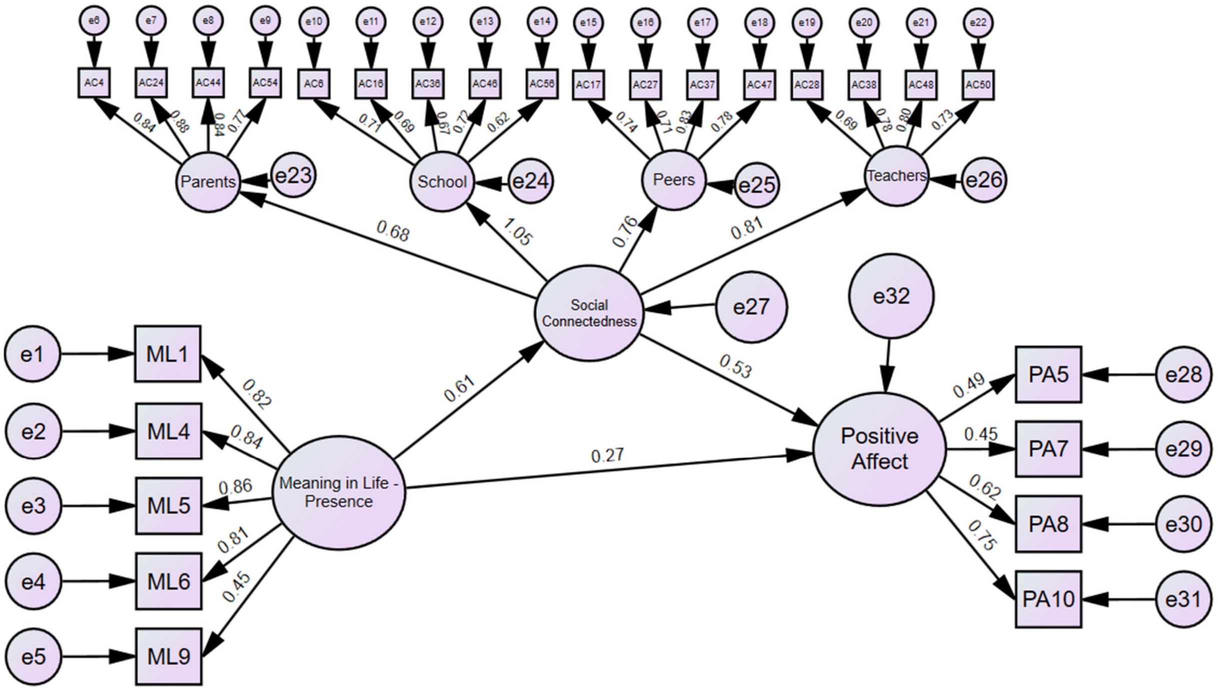

Supplementary Figure S1 Impacts of meaning in life- presence and social connectedness on positive affect

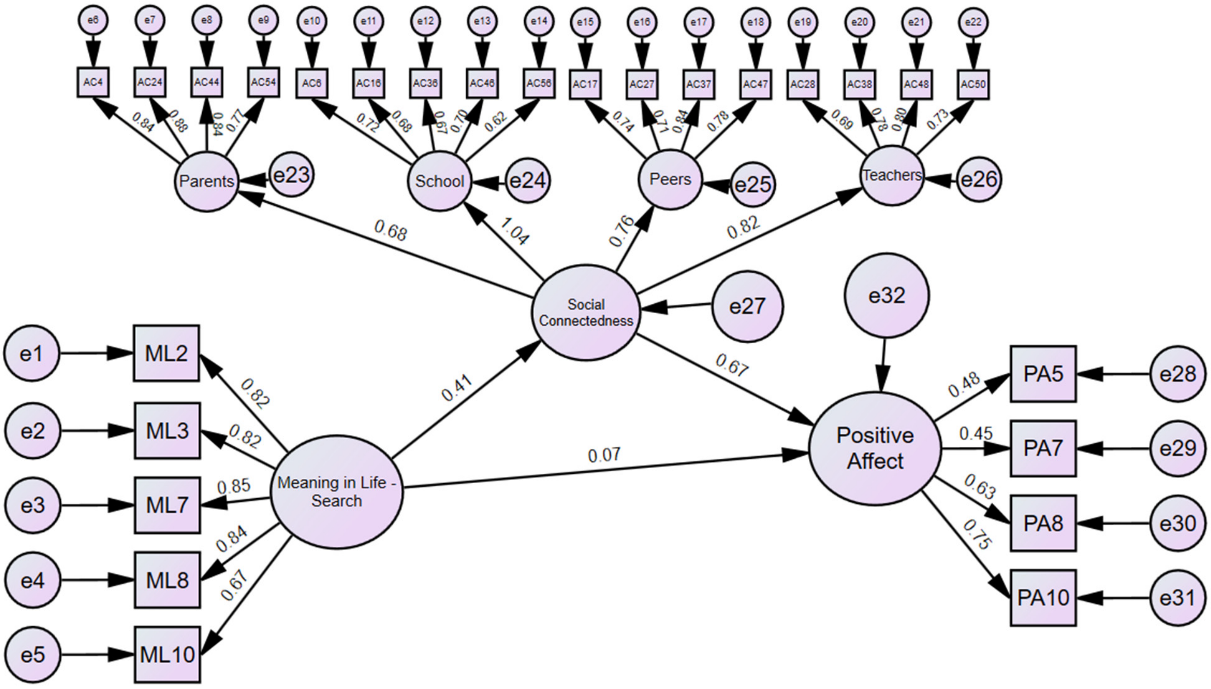

Supplementary Figure S2 Impacts of meaning in life - search and social connectedness on positive affect

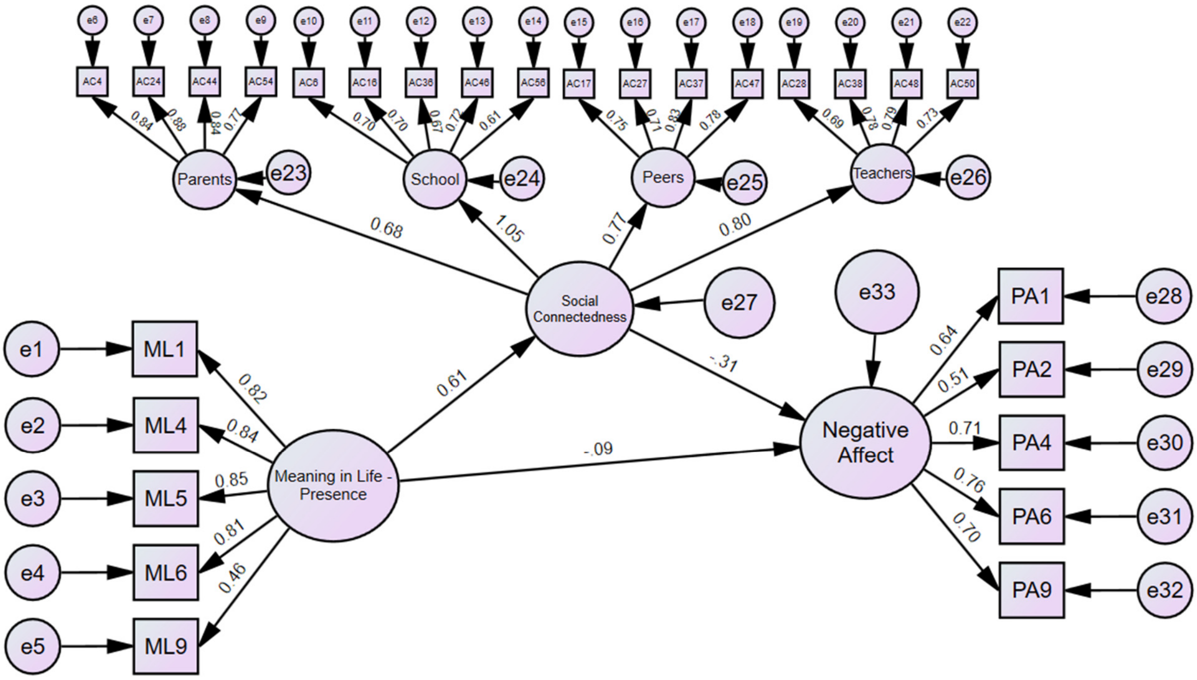

Supplementary Figure S3 Impacts of meaning in life - presence and social connectedness on negative affect

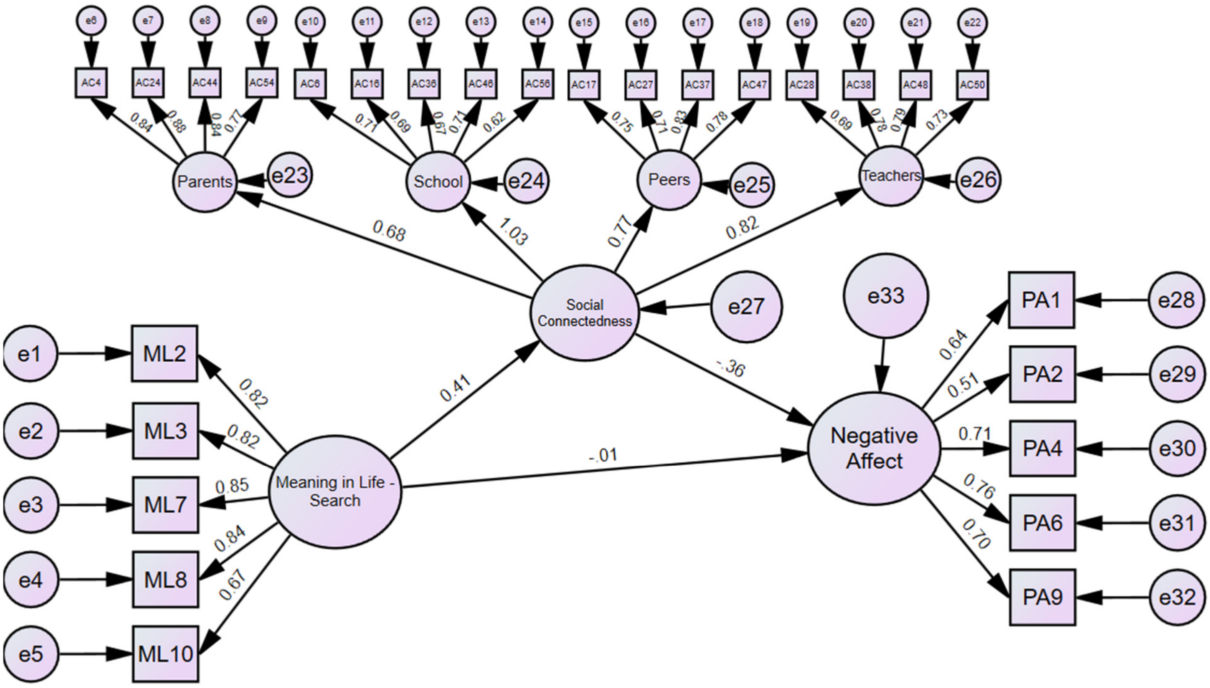

Supplementary Figure S4 Impacts of meaning in life - search and social connectedness on negative affect

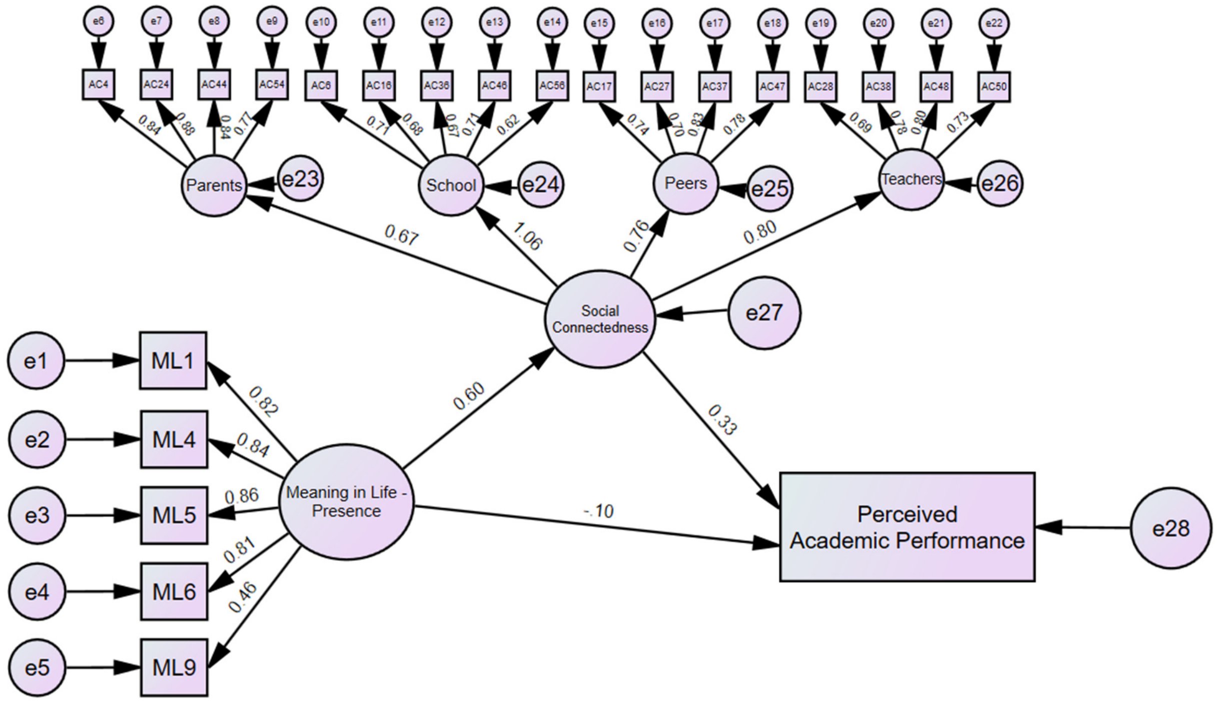

Supplementary Figure S5 Impacts of meaning in life – presence and social connectedness on perceived academic achievement

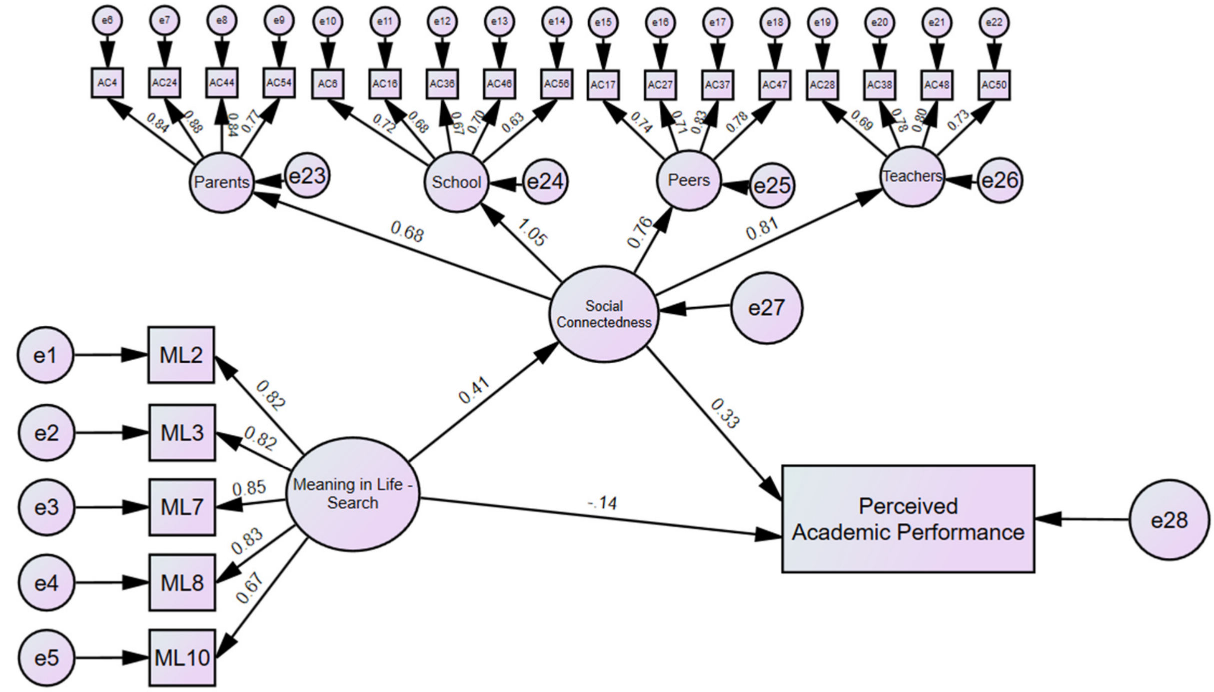

Supplementary Figure S6 Impacts of meaning in life - search and social connectedness on perceived academic achievement

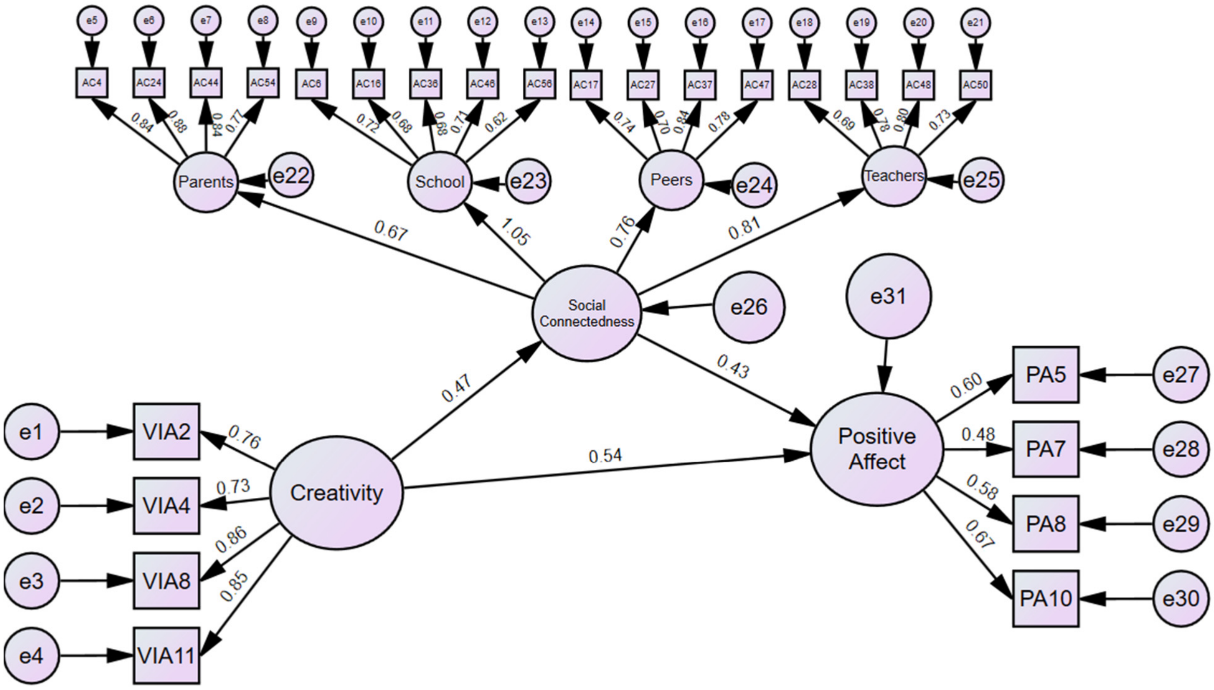

Supplementary Figure S7 Impacts of creativity and social connectedness on positive affect

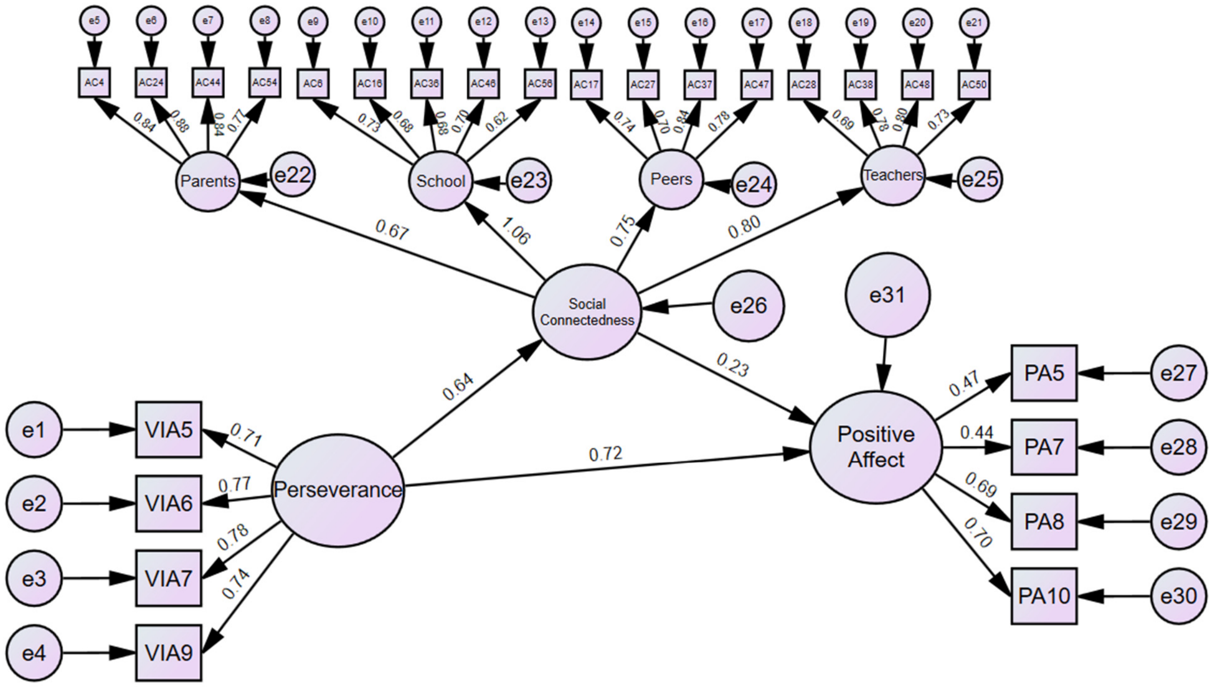

Supplementary Figure S8 Impacts of perseverance and social connectedness on positive affect

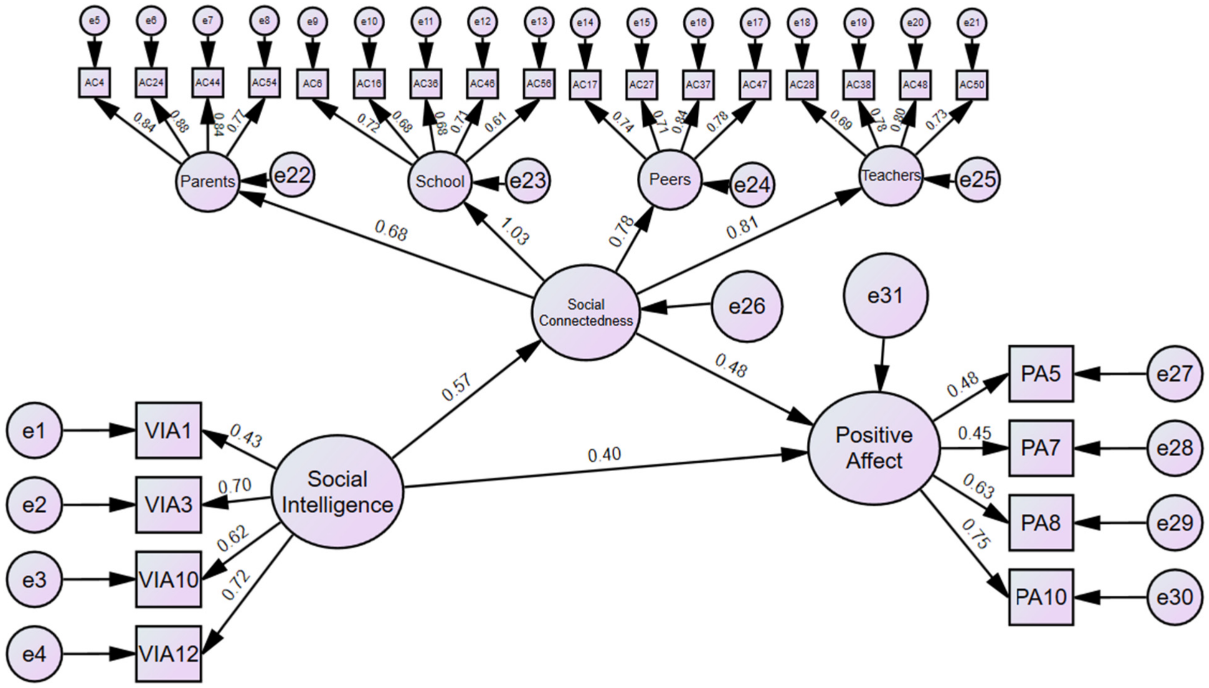

Supplementary Figure S9 Impacts of social intelligence and social connectedness on positive affect

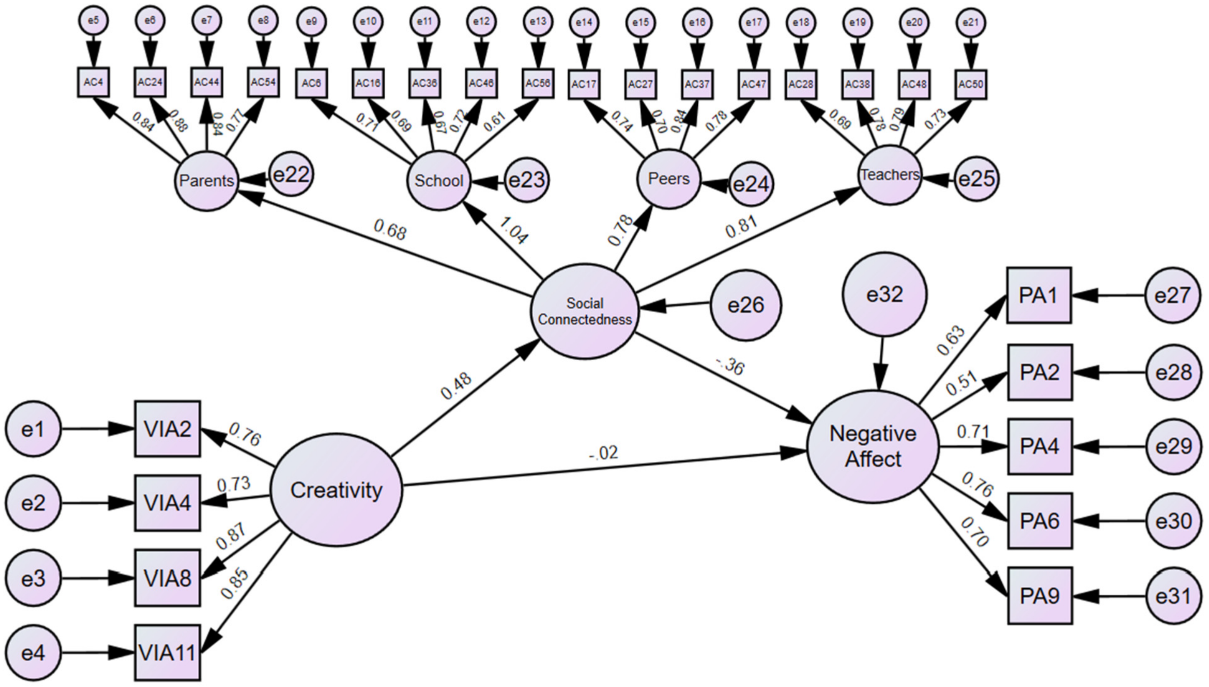

Supplementary Figure S10 Impacts of creativity and social connectedness on negative affect

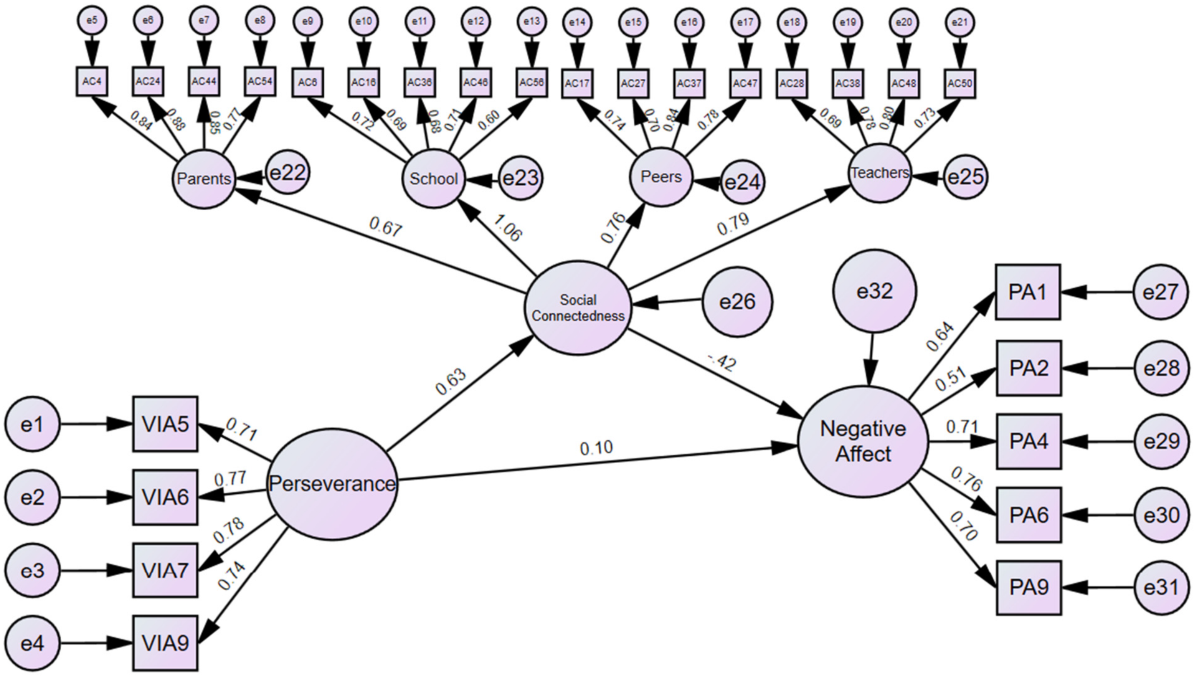

Supplementary Figure S11 Impacts of perseverance and social connectedness on negative affect

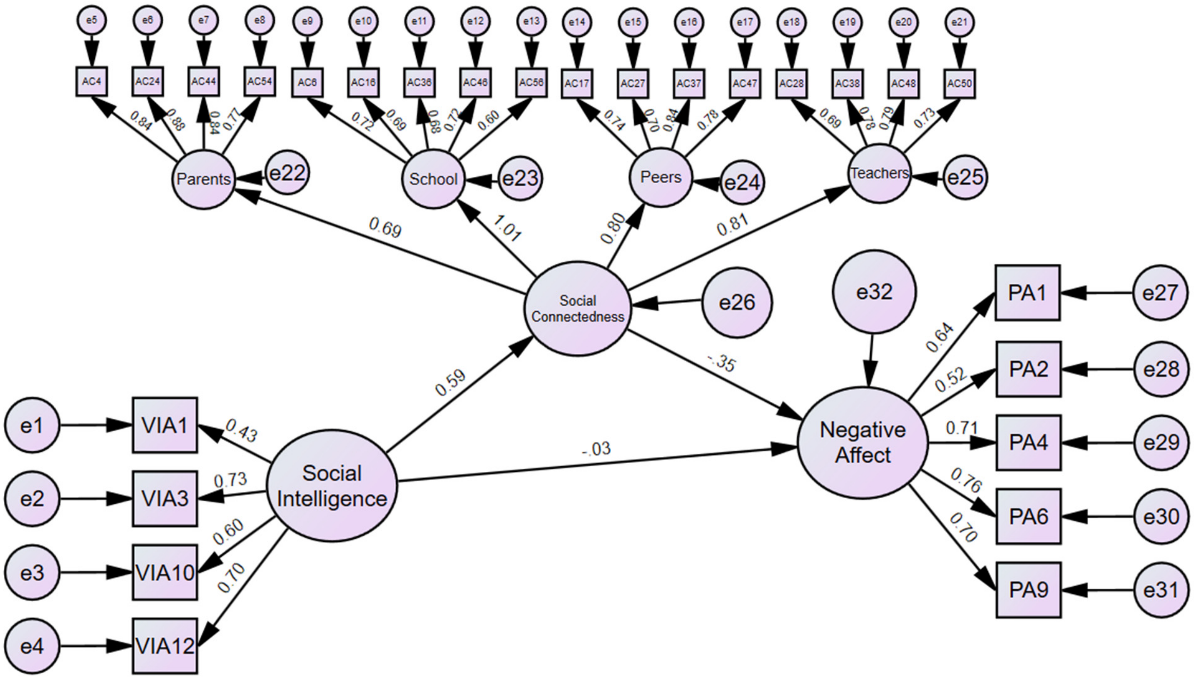

Supplementary Figure S12 Impacts of social intelligence and social connectedness on negative affect

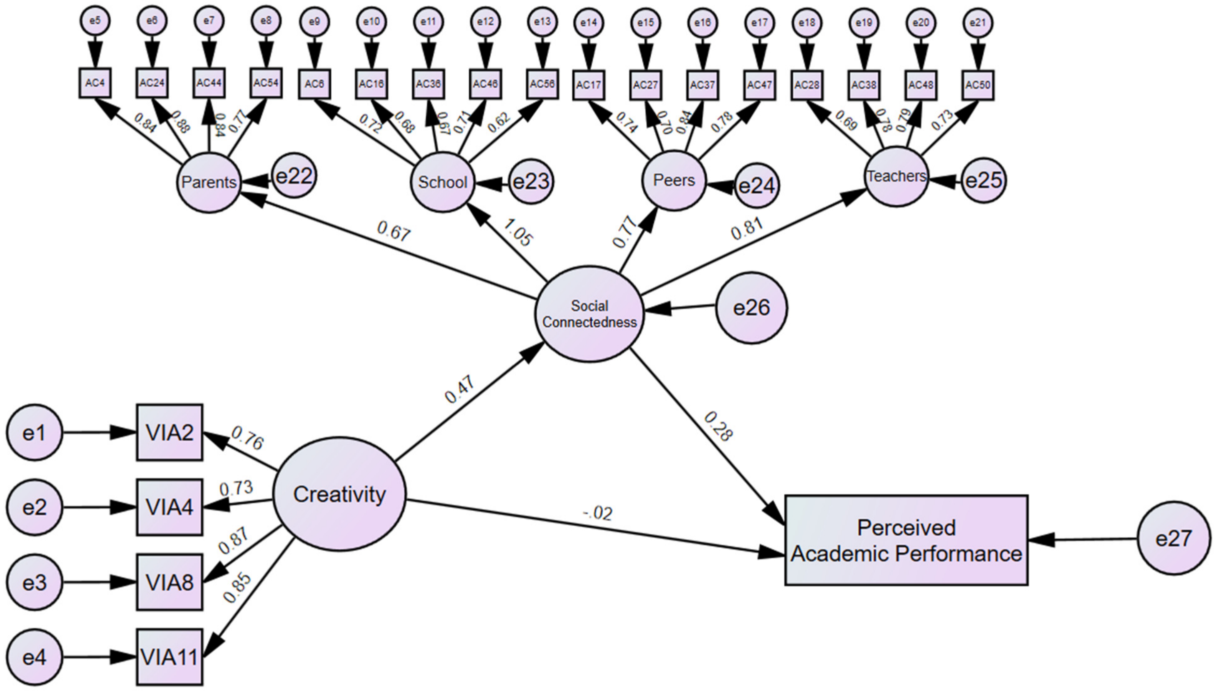

Supplementary Figure S13 Impacts of creativity and social connectedness on perceived academic achievement

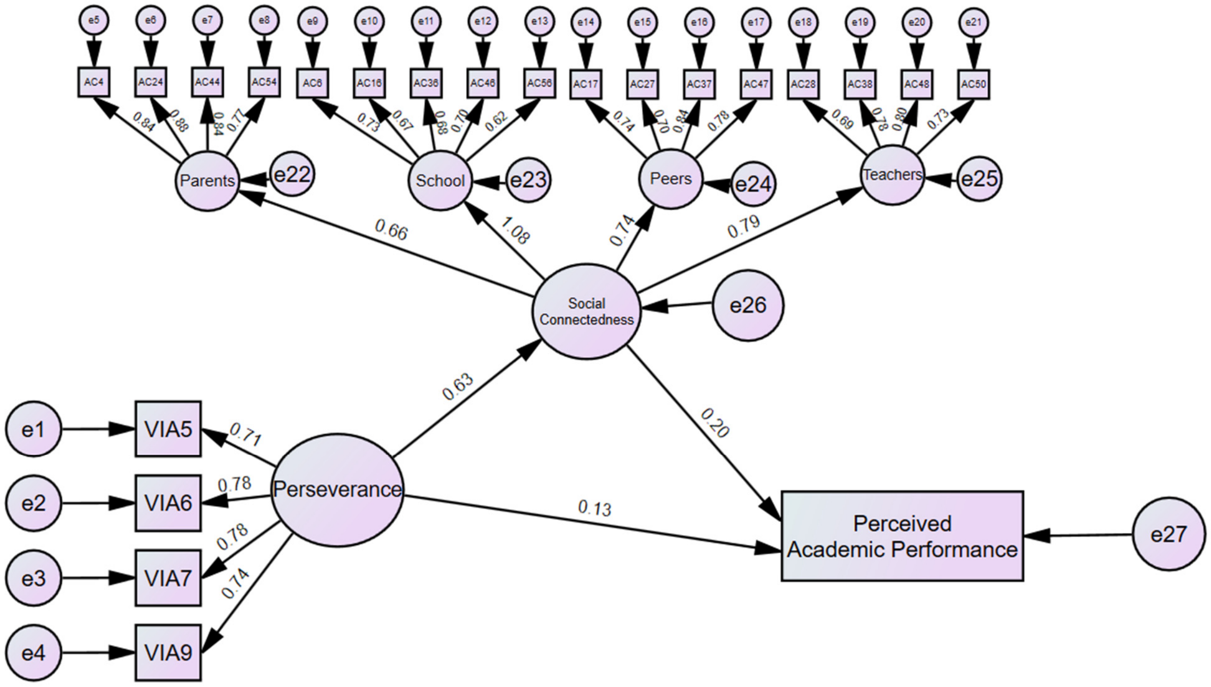

Supplementary Figure S14 Impacts of perseverance and social connectedness on perceived academic achievement

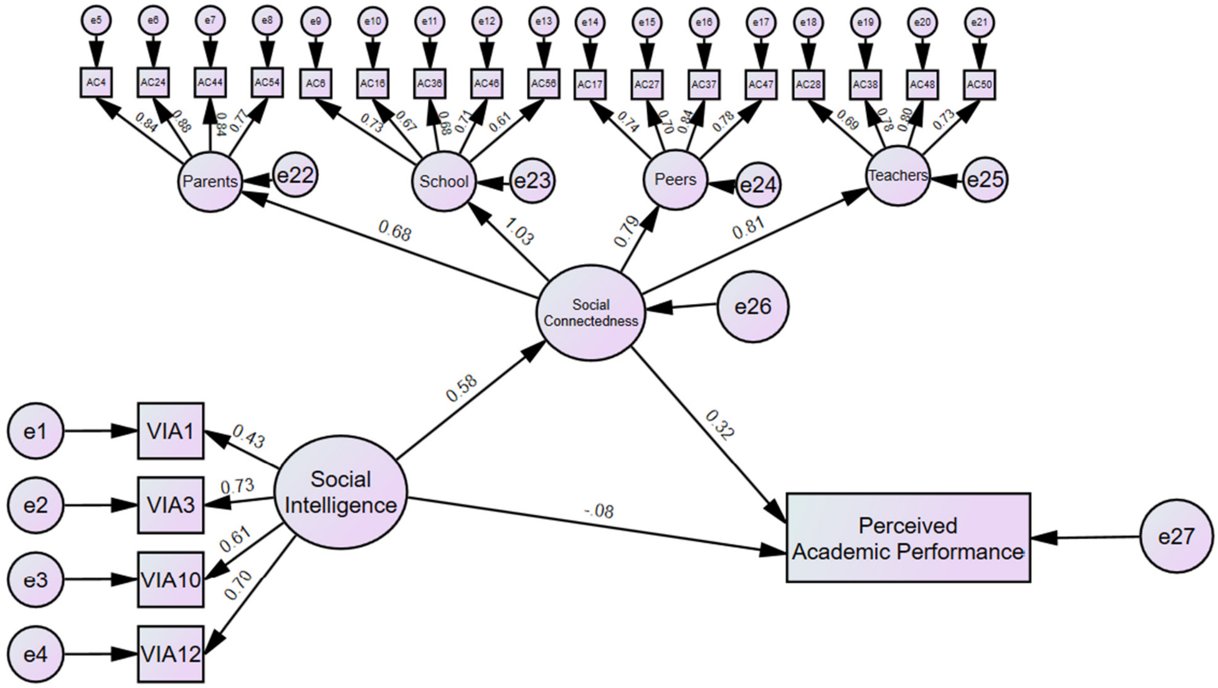

Supplementary Figure S15 Impacts of social intelligence and social connectedness on perceived academic achievement
